# Daratumumab: a first-in-class CD38 monoclonal antibody for the treatment of multiple myeloma

**DOI:** 10.1186/s13045-016-0283-0

**Published:** 2016-06-30

**Authors:** Larysa Sanchez, Yucai Wang, David S. Siegel, Michael L. Wang

**Affiliations:** Department of Medicine, Rutgers New Jersey Medical School, Newark, NJ USA; Division of Multiple Myeloma, John Theurer Cancer Center, Hackensack University Medical Center, Hackensack, NJ USA; Department of Lymphoma/Myeloma, Division of Cancer Medicine, University of Texas M. D. Anderson Cancer Center, 1515 Holcombe Blvd., Unit 429, Houston, TX 77030 USA

## Abstract

Daratumumab is a human monoclonal antibody that targets CD38, a cell surface protein that is overexpressed on multiple myeloma (MM) cells. Preclinical studies have shown that daratumumab induces MM cell death through several mechanisms, including complement-dependent cytotoxicity (CDC), antibody-dependent cell-mediated cytotoxicity (ADCC), antibody-dependent cellular phagocytosis (ADCP), and apoptosis. Given the encouraging efficacy and acceptable safety profile of daratumumab demonstrated in clinical trials, daratumumab has emerged as a novel treatment option for myeloma and became the first monoclonal antibody approved by the FDA for the treatment of MM.

## Background

Advances in the treatment of multiple myeloma (MM), particularly the development of immunomodulatory drugs (IMiDs) and proteasome inhibitors (PIs) and the use of autologous hematopoietic stem cell transplantation, have led to significant improvement in overall survival in patients with MM [1, 2]. Nevertheless, MM remains incurable and outcomes in the relapsed/refractory setting are very poor [3]. This underscores an urgent need for novel agents in the treatment of MM, especially in patients who have become refractory to currently available therapies [4]. In recent years, the introduction of monoclonal antibodies (mAbs) in MM therapy, notably mAbs targeting CD38 and SLAMF7, has been a promising step forward in improving treatment outcomes [5]. Here, we provide a brief overview of CD38 as a therapeutic target in MM and review available preclinical and clinical data on daratumumab, the first-in-class human anti-CD38 mAb approved for the treatment of MM.

## Targeting CD38 in multiple myeloma

CD38 is a 46-kDa type II transmembrane glycoprotein that is expressed on lymphoid and myeloid cells and also on non-hematopoietic tissues [6, 7]. Notably, CD38 is highly expressed on MM cells [8]. CD38 has been found to have multiple functions, including ectoenzymatic activity as well as receptor-mediated regulation of cell adhesion and signal transduction [7, 9]. The enzymatic activity of CD38 involves the conversion of nicotinamide adenine dinucleotide (NAD+) and nicotinamide adenine dinucleotide phosphate (NADP+) to cyclic adenosine diphosphate ribosyl (cADPR), ADPR, and nicotinic acid adenine dinucleotide phosphate (NAADP), substrates necessary for regulation of intracellular calcium signaling [6]. In initial studies investigating the receptor function of CD38, it was found that CD38 mediates weak cell binding to endothelium and plays a role in lymphocyte migration, as well as exhibits functional associations with surface molecules of T, B, and natural killer (NK) cells [10, 11]. The role of CD38 in cellular adhesion was further delineated with the identification of CD31 as a cell surface ligand for CD38 on endothelial cells [12]. Deaglio et al. found that CD38/CD31 interactions resulted in trans-membrane signaling characterized by calcium mobilization and cytokine secretion [12]. CD38 ligation resulting in activation of T lymphocytes was found to induce secretion of interleukin (IL)-6, granulocyte-macrophage colony-stimulating factor (GM-CSF), interferon-γ (IFN-γ), and IL-10 cytokines [13]. In other studies, CD38 ligation by agonistic mAb in NK cells was also shown to induce calcium fluxes and tyrosine phosphorylation, as well as induce NK effector function including release of IFN-γ and GM-CSF and cytotoxic responses leading to granzyme and cytokine release [14, 15]. The cellular function of CD38 and its strong expression on MM cells has made CD38 an ideal therapeutic target for the treatment of MM.

## Daratumumab in preclinical studies

Daratumumab is an immunoglobulin G1 kappa (IgG1k) human mAb that binds to a unique CD38 epitope on CD38-expressing cells with high affinity and was developed by the immunization of human immunoglobulin transgenic mice with recombinant CD38 protein [16]. de Weers et al. found that daratumumab was the only antibody in a panel of 42 human CD38-specific mAbs that triggered complement-dependent cytotoxicity (CDC) of Daudi target cells [16]. Thus, daratumumab was studied in a series of in vitro assays and was found to induce CDC in freshly isolated MM cells obtained from the bone marrow of 13 previously untreated or relapsed MM patients [16]. Furthermore, daratumumab triggered antibody-dependent cell-mediated cytotoxicity (ADCC) in CD38-expressing MM cell lines in peripheral blood mononuclear cells (PBMCs) enriched for NK cells, as well as in patient MM cells in the presence of both autologous and allogeneic effector cells [16]. Importantly, daratumumab did not induce ADCC in CD38-negative cells, confirming its specificity. Notably, daratumumab was effective at inducing both CDC and ADCC against MM cells in the presence of bone marrow stromal cells, suggesting that daratumumab is active in the bone marrow microenvironment [16]. In vivo, daratumumab exhibited high efficacy in interrupting tumor growth in mouse xenograft models [16].

In further studies investigating the mechanism of action of daratumumab, Nijhof et al. evaluated daratumumab-induced CDC or ADCC in vitro in bone marrow samples of 144 MM patients [17]. Of note, no difference was found in daratumumab-induced CDC or ADCC between newly diagnosed, relapsed/refractory, or lenalidomide- and bortezomib-refractory MM patients, suggesting that resistance to prior therapies might not affect the efficacy of daratumumab [17]. Furthermore, the investigators in this study noted a significant association between the level of CD38 expression and daratumumab-induced CDC and ADCC. Nijhof and colleagues found all-trans retinoic acid (ATRA) to upregulate CD38 expression, and pre-treatment with ATRA of patient-derived MM cells significantly increased susceptibility to daratumumab-induced CDC and ADCC in vitro [17]. Likewise, ATRA augmented daratumumab activity in a humanized MM mouse model [17].

**Fig. 1:**
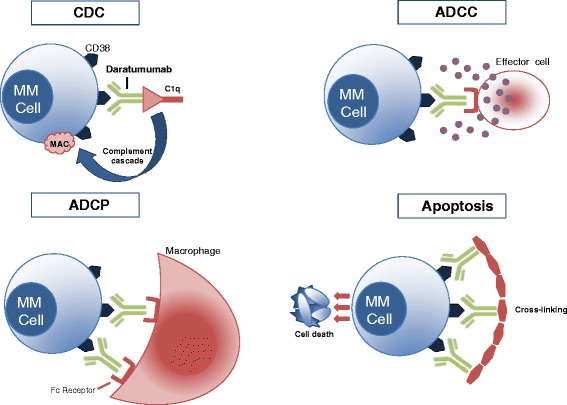
Daratumumab mechanisms of action. *Upper left*: daratumumab binds CD38, and its Fc fragment is bound by C1q, initiating complement cascade and resulting in a MAC which leads to cell lysis and death. *Upper right*: daratumumab binds CD38, and its Fc fragment is then bound by an FcR-bearing effector cell, such as a natural killer cell, leading to activation of cytotoxic processes. *Bottom left*: daratumumab binds CD38, and its Fc fragment is then bound by an FcR-bearing macrophage, inducing phagocytosis. *Bottom right*: FcR-mediated crosslinking of daratumumab induces direct cellular apoptosis. *MM cell* multiple myeloma cell, *CDC* complement-dependent cytotoxicity, *MAC* membrane attack complex, *ADCC* antibody-dependent cell-mediated cytotoxicity, *ADCP* antibody-dependent cellular phagocytosis

In addition to CDC and ADCC, daratumumab has also been shown to induce antibody-dependent cellular phagocytosis (ADCP). In a study by Overdijk and colleagues, daratumumab induced phagocytosis by human macrophages in MM cell lines in vitro observed by flow cytometry, as well as in in vivo subcutaneous and intravenous leukemic xenograft mouse models [18]. Moreover, daratumumab triggered macrophage-mediated phagocytosis ex vivo in patient-derived MM cell samples [18]. ADCP thus might be an important mechanism of action of daratumumab in the bone marrow microenvironment given that tumor-associated macrophages in the marrow have been shown to have Fc-dependent anti-tumor function [19]. Other mechanisms of daratumumab-induced cell death have been studied, finding that FcR-mediated crosslinking of daratumumab induces apoptosis of CD38-expressing tumor cells in vitro [20]. The mechanisms of action of daratumumab are shown in Fig. 1.

Preclinical studies have also investigated daratumumab together with other anti-myeloma agents, finding that daratumumab activity is enhanced in combination with agents such as lenalidomide. Given that daratumumab induces ADCC by NK cells and lenalidomide promotes NK cell activity, van der Veer et al. investigated daratumumab combined with lenalidomide on ADCC against MM cells [21]. In this study, van der Veer and colleagues used PBMCs untreated or treated with lenalidomide as effector cells against derived MM cell lines or primary MM cells obtained from patients’ bone marrow [21]. They found that pre-treatment of PBMCs with lenalidomide significantly enhanced daratumumab-induced ADCC against derived MM cell lines and primary MM cells [21]. Furthermore, the investigators treated whole bone marrow mononuclear cells of MM patients containing plasma cells with lenalidomide or daratumumab alone vs. in combination, finding that the combination of lenalidomide and daratumumab was synergistic, producing a 20 % increased effect on tumor lysis [21]. In a separate study, the synergism between lenalidomide and daratumumab on ADCC against MM cell lines was found to occur largely via NK cell activation by lenalidomide rather than direct lenalidomide effect [22]. Further synergistic effect was demonstrated in an in vivo humanized mouse model engrafted with MM cells from a lenalidomide- and bortezomib- refractory patient [22]. In this model, treatment with lenalidomide alone resulted in tumor growth compared to treatment with daratumumab alone which suppressed tumor growth, while lenalidomide in combination with daratumumab resulted in tumor reduction [22]. In another study, the combination of IPH2102, an anti-KIR antibody, with daratumumab and lenalidomide enhanced daratumumab-induced ADCC [23].

## Daratumumab in clinical trials

The promising anti-myeloma activity of daratumumab in preclinical studies led to the initiation of phase 1/2 clinical trials with daratumumab both as monotherapy and in combination regimens. Clinical trials of daratumumab with available data are summarized in Table 1.

**Table 1:** Clinical trials of daratumumab with available data

Study	NCT number (trial name)	Phase	Number	Regimen	ORR (%)	PFS rate (%) (1 year)	OS rate (%) (1 year)	Median PFS (months)
Clinical trials with daratumumab as monotherapy								
Lokhorst et al. 2015 [22]	NCT00574288 (GEN501)	1/2	30	Daratumumab (8 mg/kg)	10	–	77	2.4
			42	Daratumumab (16 mg/kg)	36	65	77	5.6
Lonial et al. 2016 [23]	NCT01985126 (SIRIUS)	2	106	Daratumumab (16 mg/kg)	29	–	65	3.7
Clinical trials with daratumumab in combination regimens								
Plesner et al. 2015 [27]	NCT01615029	1/2	32	Daratumumab (16 mg/kg) + Rd	88	–	–	–
Chari et al. 2015 [29] Mateos et al. 2015 [30]	NCT01998971	1	6	Daratumumab (16 mg/kg) + VD	100^a^	–	–	–
			11	Daratumumab (16 mg/kg) + VTD	100^b^	–	–	–
			8	Daratumumab (16 mg/kg) + VMP	100^c^	–	–	–
			77	Daratumumab (16 mg/kg) + POM-D	59^d^	–	–	–
Palumbo et al. 2016 [32]	NCT02136134 (CASTOR)	3	251	Daratumumab (16 mg/kg) + VD	83^e^	–	–	NR^e^
			247	VD	63^e^	–	–	7.16^e^

*ORR* overall response rate, *PFS* progression-free survival, *OS* overall survival, *NR* not reached, *Rd* lenalidomide and dexamethasone, *VD* bortezomib and dexamethasone, *VTD* bortezomib/thalidomide/dexamethasone, *VMP* bortezomib/melphalan/prednisone, *POM-D* pomalidomide/dexamethasone

^a^Median duration of follow-up days, 193

^b^Median duration of follow-up days, 164

^c^Median duration of follow-up days, 267

^d^ORR reported for 53 of 77 patients evaluable for efficacy; median duration of follow-up days, 148

^e^Median follow-up months, 7.4

### Daratumumab as monotherapy

The first-in-human clinical study of daratumumab (GEN501) was an open-label, multicenter, two-part, phase 1/2 clinical trial of daratumumab as a single agent in patients with relapsed or relapsed and refractory myeloma evaluating its safety, efficacy, and pharmacokinetics [24]. Thirty two patients were enrolled in part 1 (dose-escalation study), and 72 patients were enrolled in part 2 (dose-expansion study). In part 1, the patients received daratumumab at doses of 0.005 to 24 milligrams per kilogram (mg/kg) in 10 cohorts; no maximum tolerated dose (MTD) was found. In the dose-expansion study, 72 patients were enrolled, and of these, 30 patients received daratumumab at a dose of 8 mg/kg and 42 patients received daratumumab at dose of 16 mg/kg. The median age was 59 and 64 in the 8 mg/kg group and the 16 mg/kg group, respectively. The dose-expansion cohort was a heavily pre-treated population with a median of four prior therapies in both the 8 and 16 mg/kg dosing groups, and 79 % of patients were refractory to their last therapy. The overall response rate (ORR) was 36 % in the 16 mg/kg group (with 2 complete responses [CRs], 2 very good partial responses [VGPRs], and 11 partial responses [PRs]), and the median duration of response (DOR) was not reached, with a reported 12-month progression-free survival (PFS) rate of 65 % [22]. In the 8 mg/kg group, the ORR was only 10 % (with three PRs) with a median DOR of 6.9 months [24]. The investigators performed a subgroup analysis of responses based on refractoriness to prior therapy in the 16 mg/kg group: 64 % of the patients had disease refractory to both lenalidomide and bortezomib, and notably, the ORR among these double-refractory patients was 33 % [24]. The median PFS was 5.6 months in the 16 mg/kg group vs. 2.4 months in the 8 mg/kg group, and the 12-month overall survival (OS) was 77 % in both dosing groups [24]. In this heavily pre-treated and refractory patient population, daratumumab demonstrated encouraging anti-myeloma activity as a single agent as well as an acceptable safety profile. Infusion-related reactions (IRRs) occurred in 71 % of patients, most during the first infusion. The majority of IRRs were grade 1 or 2, with no treatment discontinuations due to IRR. Some of the most common grade 1 or 2 IRRs were pyrexia, influenza-like illness, fatigue, bronchospasm, nausea, and flushing. Overall, the most common non-hematologic adverse events (AEs) of all grades occurring in ≥25 % of patients in both groups were fatigue, allergic rhinitis, pyrexia, diarrhea, upper respiratory tract infection, and dyspnea. The most common hematologic AE was neutropenia, occurring in 12 % of patients in the 16 mg/kg cohort [24]. In pharmacokinetic analysis, 16 mg/kg was determined to be the lowest tested dose with consistent target saturation [24].

Daratumumab was further evaluated in an ongoing open-label, multicenter, phase II trial (SIRIUS) in patients with MM who had been treated with at least three prior lines of therapy or were refractory to both PIs and IMiDs [25]. In this study, patients were randomized to receive either daratumumab at 8 or 16 mg/kg in part 1 of the study. The 8 mg/kg cohort in part 1 did not meet criteria for dose expansion due to an ORR of only 11.1 %; thus, 16 mg/kg was the selected dose for further assessment in part 2 of the study [25]. In total, 106 patients received daratumumab 16 mg/kg in parts 1 and 2. The cohort had a median age of 63.5 years and a median of five prior lines of therapy. Moreover, 95 % of patients were refractory to the most recent PIs or IMiDs. The ORR was 29.2 % (3 stringent complete responses [sCRs], 10 VGPRs, and 18 PRs) with a median DOR of 7.4 months [25]. Notably, ORR was 29.7 % (30/101) in patients who were refractory to both PIs and IMiDs and 28.6 % (20/70) in patients who were refractory to at least three agents (bortezomib, lenalidomide, carfilzomib, or pomalidomide) [25]. The median PFS was 3.7 months, and 12-month OS was 64.8 % [25]. In this study, daratumumab demonstrated a favorable safety profile, with no treatment discontinuation due to drug-related AEs or IRR. The safety profile of both dosing cohorts was similar. In the 16 mg/kg group, the most common hematologic treatment-emergent AEs of any grade were anemia (33 %), thrombocytopenia (25 %), neutropenia (23 %), and some of the most common non-hematologic treatment-emergent AEs were fatigue, nausea, and cough. Forty two percent of patients experienced IRR, the majority of which were grade 1 or 2. The most common IRRs were nasal congestion, throat irritation, cough, dyspnea, chills, and vomiting.

In a combined analysis including 148 patients from the GEN501 and SIRIUS studies, the ORR was 31 %, and at a median follow-up of 14.8 months, the estimated OS was 19.9 months [26]. The GEN501 and SIRIUS trials demonstrated that daratumumab is active as monotherapy in patients with heavily pre-treated and relapsed/refractory myeloma (RRMM), with response rates comparing favorably to those of previous trials with pomalidomide or carfilzomib as monotherapy in RRMM [27, 28]. Importantly, daratumumab appears to maintain its anti-myeloma activity despite refractoriness to prior therapies, as seen by the encouraging ORR in patients who were refractory to both IMiDs and PIs in both studies (33 and 29.7 % in GEN501 and SIRIUS, respectively) [24, 25]. As a result of the SIRIUS trial, with findings in concordance with those of GEN501, the FDA approved daratumumab on November 16, 2015, for the treatment of refractory myeloma in patients who have received at least three previous lines of therapy, including a PI and an IMiD, or who are double refractory to a PI and IMiD.

### Daratumumab in combination regimens

The enhanced anti-myeloma activity of daratumumab in combination with other agents in preclinical trials provided rationale for investigation of daratumumab in combination regimens.

Daratumumab in combination with lenalidomide and dexamethasone in patients with relapsed or relapsed and refractory myeloma is being studied in an ongoing, phase 1/2, open-label, multicenter trial [29]. In part 1, a 3 + 3 design dose-escalation study, daratumumab was administered at doses of 2 to 16 mg/kg in combination with lenalidomide and dexamethasone until disease progression, unacceptable toxicity, or up to a maximum 24 months. Part 2 was a cohort expansion study, in which daratumumab was administered 16 mg/kg with lenalidomide and dexamethasone. Thirteen patients in total were enrolled in part 1, and MTD was not reached. Preliminary efficacy data from 20 patients presented at the 2014 American Society of Hematology (ASH) annual meeting showed marked decrease in M protein in all patients and a very encouraging ORR of 75 % [30]. The last patient was enrolled in the study in August 2014, and updated efficacy analyses including a total of 32 patients enrolled in the part 2 expansion cohort have been presented [29]. The cohort had a median of two prior lines of therapy. The ORR was 88 %, with 11 PRs, 9 VGPRs, 1 CR, and 7 sCRs [29]. The median time to first response was 1 month and median time to best response was 4.5 months, indicative of deepening responses over time [29]. The median duration of follow-up was 7.8 months, and the median DOR was not reached, with 93 % of responders remaining progression-free at time of analysis [29]. In this study, some of the most frequent reported treatment-emergent AEs were neutropenia, muscle spasms, cough, diarrhea, and fatigue. Grade 3 or 4 neutropenia was reported in 75 % of patients. Fifty six percent of patients had mild to moderate IRR, which were most commonly cough, allergic rhinitis, nausea, vomiting, and dyspnea. Of two patients who had grade 3 IRR, one patient discontinued the study due to laryngeal edema.

Daratumumab is also under investigation in combination with other MM backbone regimens in an ongoing, open-label, four-arm, multicenter, phase 1b study [31, 32]. The four arms of the study include daratumumab (at a dose of 16 mg/kg) in combination with the following regimens: bortezomib/dexamethasone (VD), bortezomib/thalidomide/dexamethasone (VTD), bortezomib/melphalan/prednisone (VMP), and pomalidomide/dexamethasone (POM-D). Inclusion criteria for each of the arms are as follows: VD and VTD arm (newly diagnosed MM, irrespective of transplant eligibility); VMP arm (newly diagnosed MM, transplant ineligible); and POM-D arm (relapsed/refractory to two or more lines of therapy including two or more consecutive cycles of lenalidomide and bortezomib) [32]. Preliminary efficacy results in newly diagnosed MM patients treated with daratumumab (DARA) in combination with VD (*n* = 6), VTD (*n* = 11), and VMP (*n* = 8) were reported by Mateos et al., with an ORR of 100 % in each arm (DARA + VD: 3 PRs, 3 VGPRs; DARA + VTD arm: 7 PRs, 2 VGPRs, and 1 CR; DARA + VMP: 4 PRs, 4 VGPRs) [32]. Median duration of follow-up days was 193, 164, and 267 days for DARA + VD, DARA + VTD, and DARA + VMP, respectively [32]. Chari et al. reported an updated efficacy analysis of the DARA + POM-D arm at the 2015 ASH annual meeting [31]. A total of 77 patients were enrolled (enrollment ongoing) at the time of the report in the POM-D arm, with median number of 3.5 prior therapies. Of the cohort, 65 % were refractory to both a PI and IMiD. Of the 77 patients enrolled, 53 were evaluable for efficacy. The ORR was 58.5 %, with 3 sCRs, 1 CR, 12 VGPRs, and 15 PRs [31]. After a median follow-up of 148 days, 4/31 responders progressed [31]. Notably, the combination of daratumumab with POM-D exhibited remarkable efficacy among 40 evaluable double-refractory patients, with a 57.5 % ORR [31]. In the POM-D arm, 61 % of patients experienced IRR, which were most commonly chills, cough, and dyspnea. Otherwise, no significant additional toxicity was noted when DARA was added to POM-D. Some of the most common AEs of any grade were neutropenia, anemia, fatigue, cough, nausea, dyspnea, and diarrhea. Grade 3 or 4 AEs occurring in >10 % of patients were all hematologic toxicities (neutropenia [50.6 %], anemia [20.8 %], leukopenia [15.6 %], and thrombocytopenia [10.4 %]).

The remarkable efficacy of DARA in combination with both lenalidomide/dexamethasone and POM-D in RRMM and in combination with backbone regimens in newly diagnosed MM has provided rationale for initiation of phase III trials. MMY3003 (POLLUX) and MMY3004 (CASTOR) are currently ongoing randomized, open-label, multicenter, phase III trials for patients with relapsed or refractory MM, in which MMY3003 will compare the efficacy of daratumumab in combination with lenalidomide and dexamethasone vs. lenalidomide and dexamethasone alone, and MMY3004 will compare the efficacy of daratumumab in combination with bortezomib and dexamethasone vs. bortezomib and dexamethasone alone [33]. An interim analysis of the phase III CASTOR (MMY3004) trial presented at the 2016 American Society of Clinical Oncology annual meeting showed that daratumumab significantly improved ORR, PFS, and time to progression (TTP) in combination with bortezomib and dexamethasone [34]. A total of 498 patients with relapsed or refractory myeloma were randomized to receive daratumumab in combination with bortezomib and dexamethasone or bortezomib and dexamethasone alone. The ORR was 83 % in patients who received daratumumab, bortezomib, and dexamethasone compared to 63 % in patients who received bortezomib and dexamethasone alone (*p* < 0.0001) [34]. With a median follow-up of 7.4 months, median PFS and TTP in the daratumumab arm was not reached, whereas the median PFS and TTP in the bortezomib and dexamethasone arm was 7.16 and 7.29 months, respectively [34]. The combination of daratumumab, bortezomib, and dexamethasone significantly improved both PFS (HR 0.39; 95 % CI, 0.28–0.53; *p* < 0.0001) and TTP (HR 0.30; 95 % CI, 0.21–0.43; p < 0.001) compared to bortezomib and dexamethasone alone [34]. IRR occurred in 45 % of patients, and the most common grade 3/4 AEs were hematologic toxicities, with higher rates of thrombocytopenia (45 vs. 33 %) and neutropenia (13 vs. 4 %) occurring in the daratumumab arm.

The efficacy of daratumumab in newly diagnosed MM is also to be studied in a randomized, open-label, multicenter, phase III trial (Alcyone) that will compare VMP (bortezomib, melphalan, prednisone) vs. DARA + VMP in patients with newly diagnosed, previously untreated MM who are ineligible for high-dose therapy with stem-cell transplantation [35]. In another randomized, phase III trial (Cassiopeia), newly diagnosed transplant-eligible patients will be treated with VTD with or without daratumumab as induction therapy prior to transplant followed by VTD with or without daratumumab as consolidation therapy and then re-randomized to daratumumab maintenance therapy vs. observation. The ongoing clinical trials of daratumumab are listed in Table 2.

**Table 2:** Ongoing clinical trials of daratumumab in multiple myeloma

NCT number	Title	Phase	Number	Recruitment
NCT02116569	A study of daratumumab in Japanese participants with relapsed or refractory multiple myeloma	1	9	Completed
NCT02497378	A study of JNJ-54767414 (daratumumab) in combination with bortezomib and dexamethasone (D-Vd) in Japanese participants with relapsed or refractory multiple myeloma	1	6	Recruiting
NCT02519452	A study of daratumumab with the addition of recombinant human hyaluronidase (rHuPH20) for the treatment of participants with relapsed or refractory multiple myeloma	1	128	Recruiting
NCT02626481	Study of daratumumab in combination with dexamethasone in resistant or refractory multiple myeloma	2	64	Recruiting
NCT02316106	A study to evaluate 3 dose schedules of daratumumab in participants with smoldering multiple myeloma	2	120	Recruiting
NCT02419118	Monoclonal antibodies for treatment of multiple myeloma. Emphasis on the CD38 antibody daratumumab	2/3	50	Recruiting
NCT02076009	A study comparing daratumumab, lenalidomide, and dexamethasone with lenalidomide and dexamethasone in relapsed or refractory multiple myeloma	3	571	Active, not recruiting
NCT02136134	Addition of daratumumab to combination of bortezomib and dexamethasone in participants with relapsed or refractory multiple myeloma	3	497	Active, not recruiting
NCT02541383	A study to evaluate daratumumab in transplant eligible participants with previously *untreated* multiple myeloma (Cassiopeia)	3	1080	Recruiting
NCT02252172	Study comparing daratumumab, lenalidomide, and dexamethasone with lenalidomide and dexamethasone in participants with previously *untreated* multiple myeloma	3	730	Recruiting
NCT02195479	A study of combination of daratumumab and Velcade (bortezomib) melphalan-prednisone (DVMP) compared to Velcade melphalan-prednisone (VMP) in participants with previously *untreated* multiple myeloma	3	700	Recruiting

### Daratumumab interference with laboratory testing

Daratumumab has been found to interfere with serum protein electrophoresis (SPEP) and immunofixation electrophoresis (IFE) assays due to co-migration of daratumumab with patients’ M protein, particularly with IgGk M proteins, which impedes accurate quantification of endogenous M protein [36]. This poses a clinical problem given SPEP and IFE assays are necessary for assessment of disease response in MM. A clinical assay, the daratumumab IFE reflex assay (DIRA), has been developed to mitigate daratumumab interference with IFE [36]. DIRA uses a mouse anti-daratumumab antibody that binds to daratumumab and shifts the migration of daratumumab away from the M protein [36]. The specificity, reproducibility, and concordance of DIRA to distinguish daratumumab mAb from endogenous M protein were validated in a separate study [37]. The DIRA assay has been utilized in clinical trials of daratumumab to confirm disease response [24].

It has also been observed that the plasma of daratumumab-treated patients demonstrates positive antibody screens and panreactivity on blood compatibility testing as a result of daratumumab binding to CD38 on reagent red blood cells (RBCs) [38]. This finding is clinically relevant as MM patients undergoing treatment with daratumumab may, at some point, require blood transfusions and accurate detection of alloantibodies in blood compatibility testing is imperative in preventing blood transfusion reactions. Techniques by which to ameliorate daratumumab interference with blood compatibility testing are being investigated. Treating reagent RBCs with dithiothreitol (DTT), which denatures CD38, or the addition of neutralizing agents such as anti-idiotype antibodies against daratumumab or recombinant soluble CD38 to patients’ plasma has been found to inhibit blood compatibility testing interference [38, 39]. The results of a multicenter, international validation study on the use of DTT to resolve daratumumab interference with blood compatibility testing were presented at the 2015 ASH annual meeting [40]. In this study, participating blood banks received two unknown plasma samples, one sample with daratumumab and the other sample with daratumumab and an unknown clinically significant RBC antibody. The blood banks then performed standard antibody screening as well as antibody screening with DTT-treated RBCs. Of 23 participating sites, all sites reported daratumumab interference with standard antibody screening (gel, tube, or solid phase testing) but no interference using DTT-treated RBCs [40]. Using the DTT method, all 23 sites were able to correctly identify the unknown RBC antibody [40]. As DTT is known to also denature Kell antigens, study investigators recommend that K negative blood be administered when using the DTT method [38, 40]. As the use of therapeutic antibodies for the treatment of MM increases, clinicians must remain mindful of potential laboratory interference with daratumumab as well as other mAbs.

## Conclusions

Daratumumab has been well tolerated and has demonstrated encouraging response rates in clinical trials both as a single agent and in combination regimens in the relapsed/refractory setting [24, 25, 29, 31]. Moreover, daratumumab in combination regimens has maintained a favorable safety profile without significant increase in toxicities [29, 31, 34]. Clinical trials with other CD38-targeting mAbs isatuximab (or SAR650984) and MOR202 have also shown promising results [41–43]. Phase III trials of daratumumab both in the relapsed/refractory setting as well as in newly diagnosed patients will help to elucidate the role of daratumumab in the treatment of MM. Given the remarkable efficacy that has been seen with daratumumab in early clinical trials, daratumumab as well as other mAbs are likely to change the landscape of myeloma treatment.

## Abbreviations

ADCC, antibody-dependent cell-mediated cytotoxicity; ADCP, antibody-dependent cellular phagocytosis; AE, adverse event; ATRA, all-trans retinoic acid; CDC, complement-dependent cytotoxicity; CR, complete response; DARA, daratumumab; DIRA, daratumumab IFE reflex assay; DOR, duration of response; DTT, dithiothreitol; GM-CSF, granulocyte-macrophage colony-stimulating factor; IFE, immunofixation electrophoresis; IMiD, immunomodulatory drug; IRRs, infusion-related reactions; mAbs, monoclonal antibodies; MM, multiple myeloma; MTD, maximum tolerated dose; NK, natural killer; ORR, overall response rate; OS, overall survival; PBMC, peripheral blood mononuclear cell; PFS, progression-free survival; PIs, proteasome inhibitors; POM-D, pomalidomide/dexamethasone; PR, partial response; RBC, red blood cell; sCR, stringent complete response; SPEP, serum protein electrophoresis; VD, bortezomib/dexamethasone; VGPR, very good partial response; VMP, bortezomib/melphalan/prednisone; VTD, bortezomib/thalidomide/dexamethasone
